# The impact of institutionalization and adoption on ADHD diagnosed children

**DOI:** 10.1192/j.eurpsy.2025.1134

**Published:** 2025-08-26

**Authors:** T. M. Gómez Lezcano, C. Fernandez Natal, P. Campos Abraham, M. M. Gonzalvo Navarro, M. B. San José, M. V. Sánchez, M. García López

**Affiliations:** 1 Psychiatry Department, Hospital Del Henares, Coslada, Madrid, Spain

## Abstract

**Introduction:**

Adoption is a process which aspires to protect the children and provide them a secure home. Nevertheless these minors present very often in their new environment a wide range of different difficulties, that enhances them to adapt in their personal and academic spaces. ADHD is one of the most prevalent neurodevelopment diseases among adopted children (Mulas et al., 2016; Doom et al., 2016).

**Objectives:**

The objective of this review is to shed light on the association between ADHD and adoption.

**Methods:**

A literature review has been carried out of scientific studies on the prevalence and risk factors for developing ADHD among adopted children from different countries of origin. Systematic reviews of papers and studies on new developments in the etiology of ADHD were also consulted.

**Results:**

The prevalence of ADHD in adopted children is estimated to be around 25-50% (Mulas et al., 2013; Enriquezet. al., 2017). However, this percentage varies considerably depending on the origin of the children (Eastern Europe (de Maat et al., 2018), versus China (van Ginkel et al. 2016)). However, adopted children are more easily referred to a mental health specialist, as a consequence of the fact that adoptive parents are often particularly focused on the adaptation of these children (Sánchez et al., 2012).

The postnatal environment of adopted and institutionalized childrenchildren is often marked physical, emotional, sexual or other types of abuse (Doom et al., 2016). The quality of these orphanages and foster homes contributes to the lack of stimuli that children should receive in the first 2 to 3 years of life (Mullas et al., 2015). Consequently, this social and emotional deprivation among adopted children has been related to attachment disorders and ADHD (Elovainio et al., 2015).

On the other hand, other pre- and perinatal risk factors that influence the etiology of this disorder are especially prevalent in adopted children: premature birth, low birth weight and exposure to alcohol during pregnancy (Macke et al., 2020).

The role of culture as a possible protective factor is supported by research, with immigrants showing lower rates of ADD than adopted children of the same age range (Tan et al., 2016).

Finally, early foster care in adoptive families and the promotion of family bonding and appropriate parenting strategies can help adopted children improve their regulatory skills and other typical ADHD symptoms (Barca et al.,2017).

**Conclusions:**

The deprivation of affective care and the lacking opportunity to establish a relationship with a main caregiver experienced by institutionalized children can lead to the expression of this disease in genetically predisposed individuals. These fenomenon reveals the importance of connection to family and culture to promote better mental health.

**Disclosure of Interest:**

None Declared

